# Competing Easy-Axis Anisotropies Impacting Magnetic Tunnel Junction-Based Molecular Spintronics Devices (MTJMSDs)

**DOI:** 10.3390/ijms232214476

**Published:** 2022-11-21

**Authors:** Bishnu R. Dahal, Andrew Grizzle, Christopher D’Angelo, Vincent Lamberti, Pawan Tyagi

**Affiliations:** 1 Center for Nanotechnology Research and Education, Mechanical Engineering, University of the District of Columbia, Washington, DC 20008, USA; 2 Y-12 National Security Complex, Oak Ridge, TN 37830, USA

**Keywords:** magnetic tunnel junctions, single-molecule magnets, Monte Carlo simulations, spintronics, anisotropy

## Abstract

Molecular spintronics devices (MSDs) attempt to harness molecules’ quantum state, size, and configurable attributes for application in computer devices—a quest that began more than 70 years ago. In the vast number of theoretical studies and limited experimental attempts, MSDs have been found to be suitable for application in memory devices and futuristic quantum computers. MSDs have recently also exhibited intriguing spin photovoltaic-like phenomena, signaling their potential application in cost-effective and novel solar cell technologies. The molecular spintronics field’s major challenge is the lack of mass-fabrication methods producing robust magnetic molecule connections with magnetic electrodes of different anisotropies. Another main challenge is the limitations of conventional theoretical methods for understanding experimental results and designing new devices. Magnetic tunnel junction-based molecular spintronics devices (MTJMSDs) are designed by covalently connecting paramagnetic molecules across an insulating tunneling barrier. The insulating tunneling barrier serves as a mechanical spacer between two ferromagnetic (FM) electrodes of tailorable magnetic anisotropies to allow molecules to undergo many intriguing phenomena. Our experimental studies showed that the paramagnetic molecules could produce strong antiferromagnetic coupling between two FM electrodes, leading to a dramatic large-scale impact on the magnetic electrode itself. Recently, we showed that the Monte Carlo Simulation (MCS) was effective in providing plausible insights into the observation of unusual magnetic domains based on the role of single easy-axis magnetic anisotropy. Here, we experimentally show that the response of a paramagnetic molecule is dramatically different when connected to FM electrodes of different easy-axis anisotropies. Motivated by our experimental studies, here, we report on an MCS study investigating the impact of the simultaneous presence of two easy-axis anisotropies on MTJMSD equilibrium properties. In-plane easy-axis anisotropy produced multiple magnetic phases of opposite spins. The multiple magnetic phases vanished at higher thermal energy, but the MTJMSD still maintained a higher magnetic moment because of anisotropy. The out-of-plane easy-axis anisotropy caused a dominant magnetic phase in the FM electrode rather than multiple magnetic phases. The simultaneous application of equal-magnitude in-plane and out-of-plane easy-axis anisotropies on the same electrode negated the anisotropy effect. Our experimental and MCS study provides insights for designing and understanding new spintronics-based devices.

## 1. Introduction

It has been over a decade since electron spin debuted in the semiconductor device industry [1,2,3]. The new field of electronics, called spintronics, harnesses the intrinsic spin of an electron and its associated magnetic moment along with its electronic charge [4]. Spintronics has already revolutionized computer memory devices [5]. Spintronics possesses inestimable potential for futuristic computer technology, including the development of quantum computers [6] and combining logic and memory in the same device [3,7]. A significant limitation of the emerging technology is that it is based on limited traditional materials such as inorganic metals and semiconductors. Utilization of the ferromagnetic metal is essential because of the high Curie temperature required for its commercially useful applications [2,8]. Alloying magnetic materials and stacking multiple magnetic layers offer the possibility of obtaining various magnetic properties [9,10].

A new spintronics field is emerging that combines the quantum properties of the mass-producible molecule as the device element [11,12,13] between two ferromagnetic electrodes [14,15]. Connecting molecules between two ferromagnetic electrodes opens the flood gate for innovations. Interestingly, commercially successful magnetic tunnel junction (MTJ) technology comes very close to the concept of connecting ferromagnetic electrodes with molecules as active transport channels. However, unlike MTJs, which only rely on magnesium oxide as an insulator [10] due to MTJs desirable switching attributes [1,9], molecule-based spintronics has billions of types of molecules that can be included as spin channels. Molecules can be designed with useful optical, magnetic, and electrical properties. Most importantly, a desirable molecule can be mass-produced to sub-angstrom level structural precision [16,17,18,19].

Molecular spintronics devices (MSDs) can overcome the miniaturization limits and heating issues associated with existing computer technology [20]. However, due to the nanoscale size of the molecules (∼1 nm), it is difficult to keep the molecular dimension robust and maintain a reproducible gap between the two ferromagnetic leads [21]. To avoid these difficulties, we developed a new approach to making magnetic tunnel junction-based molecular spintronics devices (MTJMSDs). To produce an MTJMSD, the molecular channels were bridged across the insulator of an MTJ testbed with exposed side edges of the FM electrodes. MTJMSD properties and their applications are highly influenced by ferromagnetic electrodes’ physical properties, such as their various anisotropies, thermal energy, coupling of the ferromagnetic electrode atoms of two electrodes via magnetic molecules, etc. [22]. Interestingly, we observed a remarkable difference between the on and off states in MTJMSDs [23]. However, this observation was transient and insufficient to yield repeatable switching at room temperature.

Under the aspiration of making bistable memory devices, we experimentally realized an MTJMSD by including two multi-layered magnetic electrodes with different magnetic properties, deposited via sputtering process. Prior literature shows that the simple addition of seed layers and simply altering the sequence of thin ferromagnetic layers dramatically impact the magnetization properties of electrodes and devices [24,25,26,27,28]. Here, we discuss experimental studies showing the impacts of various magnetic electrodes on MTJMSDs. Cross-junction-shaped MTJMSDs designed for conducting transport studies possessed long ferromagnetic electrodes. Long ferromagnetic electrodes enable the connection of molecule–ferromagnetic electrode interfaces with the outer world for transport and device attributes. However, understanding the impact of the interaction between paramagnetic molecules and long multilayered ferromagnetic electrodes was experimentally challenging. The challenge of understanding MSD is harder when ferromagnetic electrodes possess different magnetic anisotropies. It is a daunting task to understand the overall device properties of MTJMSDs experimentally when individual in-plane and out-of-plane easy-axis anisotropies are operating. Here, we also present our Monte Carlo simulations (MCSs) of an MTJMSD with extended electrodes of variable anisotropies. In the MCS study, we systematically applied the in-plane and out-of-plane anisotropies individually and together to gain an atomistic understanding of the resultant equilibrium properties.

## 2. Results and Discussions

**Figure 1:**
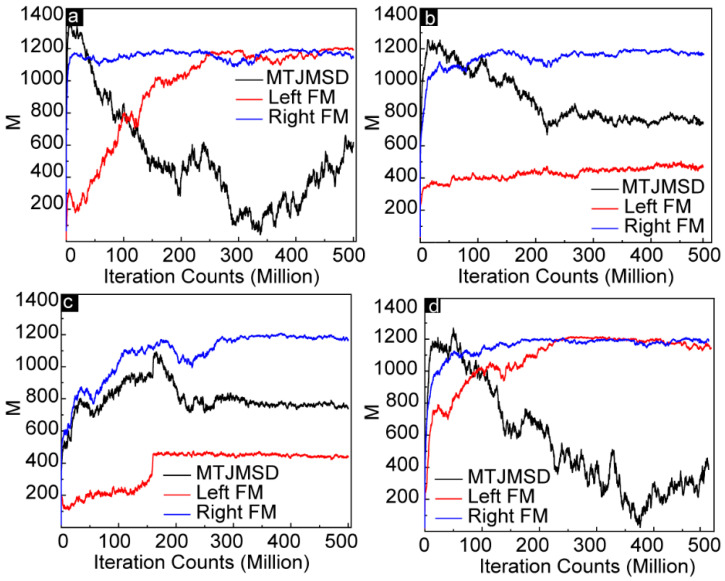
Temporal evolution of the MTJMSD and two electrodes, measured by monitoring magnetic moment as a function of iteration counts (equivalent to simulation time) for (**a**) *A_Lx_* = 0 and *A_Ly_* = 0, (**b**) *A_Lx_* = 0 and *A_Ly_* = 0.5, (**c**) *A_Lx_* = 0.5 and *A_Ly_* = 0, and (**d**) *A_Lx_* = 1 and *A_Ly_* = 1.

The first step in exploring the effect of anisotropy on MTJMSD focused on understanding the evolution of the equilibrium state from perturbed states. For this objective, we explored the evolution of MTJMSD over time for combinations of *A_Lx_* and *A_Ly_.* Figure 1 shows the impact of anisotropies on the overall magnetic properties of MTJMSD during the energy minimization of the MCS (magnetic moment vs. iteration counts) with given in-plane and out-of-plane easy-axis anisotropies. Temporal evolutions were recorded at *kT* = 0.1. Figure 1a shows the variation in the magnetic moment of the MTJMSD as a function of iteration counts when there were no anisotropies on the left ferromagnetic electrode. Based on the dimensions of the MTJMSD used during the MCS, the left and right ferromagnetic electrodes could attain a maximum magnetic moment magnitude of 1250. At the same time, the MTJMSD’s maximum magnetic moment settled around 2516 (1250 for each ferromagnetic electrode and 16 for molecules). It is noteworthy that we kept the right electrode isotropic during the simulation. As a result, the total magnetic moment of the right FM electrode was always close to its maximum value of ~1200. When *A_Lx_* = 0 and *A_Ly_* = 0 the magnetic moment of the left electrode started to increase quasi-linearly with the iteration counts before it saturated at around 250 million iterations. The magnetic moment of the left electrode saturated to its maximum value of ~1150. In the absence of anisotropies, the antiferromagnetic coupling provided by the Heisenberg coupling of the left and right electrodes with the molecules was the dominating factor. The molecule coupling with electrodes was *J_mR_* = 1 and *J_mL_* = −1, respectively. The total magnetic moment of the MTJMSD was always lower than that of the left and right electrodes due to the opposite magnetic spins of the left and right FM electrodes (Figure 1a). When *A_Lx_* = 0 but *A_Ly_* = 0.5, the in-plane anisotropy was forced to align the magnetic spins of the left ferromagnetic electrode and overcame the effect of *J_mL_* (Figure 1b). It was also observed that *A_Ly_* caused the magnetic moment to particularly align in the spin direction opposing *J_mL_*.

As a result, the magnetic moment of the left electrode was decreased, but that of the MTJMSD increased. The impact of out-of-plane anisotropy and the competing effect of in-plane and out-of-plane anisotropy are described in Figure 1c,d, respectively. When *A_Lx_* = 0.5 and *A_Ly_* = 0 (Figure 1c), the impact of out-of-plane anisotropy was somewhat similar as when provided with an equal magnitude of in-plane anisotropy (Figure 1d).

A notable observation occurred at around 175M iteration counts. At this stage, a sudden jump in the magnetic moment of the left FM electrode was observed (Figure 1c). The sudden jump in the magnetic moment of the left FM electrode was due to the formation of the dominant magnetic phase of the same spin orientations due to the out-of-plane easy-axis anisotropy, which will be further discussed in this manuscript. The magnetic moment saturated close to ~425, immediately after the jump of the magnetic moment. With the application of in-plane and out-of-plane anisotropies on the same left ferromagnetic electrode, we observed that the effects of the anisotropies started to annihilate each other (Figure 1d). We refer to annihilation as “competing impact” in this report. The competing impact of anisotropies produced a high value of magnetic moments by aligning all the magnetic spins of the atoms of the left electrode. However, the orientation of the magnetic spins of the left FM electrode was opposite to that of the isotropic right electrode due to strong molecule-induced antiferromagnetic coupling. Therefore, the total magnetic moment of the MTJMSD was observed to be smaller than that of the left and right electrodes. When the magnetic moments of the left and right electrodes were close to equal, but the magnetic spins of the left and right FM electrodes were opposite (~325 M iteration counts), the total magnetic moment of the MTJMSD was almost zero, as shown in Figure 1d. The overall magnetic moment of the MTJMSD was similar when anisotropies were not applied to the left FM electrode (Figure 1a) or when the FM electrode had an equal magnitude of in-plane and out-of-plane anisotropies (Figure 1d). The prior case happened due to antiferromagnetic Heisenberg coupling of the left and right FM electrodes with paramagnetic molecules. In comparison, the latter case was due to the competing effect of in-plane and out-of-plane anisotropies on the left FM electrode.

**Figure 2:**
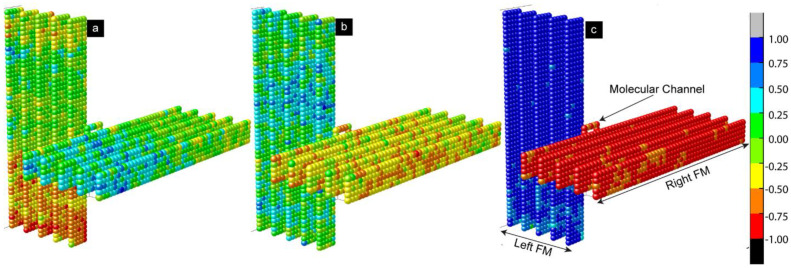
Spatial 3D lattice model of the MTJMSD measured in the equilibrium state at the end of the simulations at *kT* = 0.1 and *A_Lx_* = *A_Ly_* = 0 along (**a**) spin direction on the *X*-axis, (**b**) spin direction on the *Y*-axis, and (**c**) spin direction on the z-axis.

**Figure 3:**
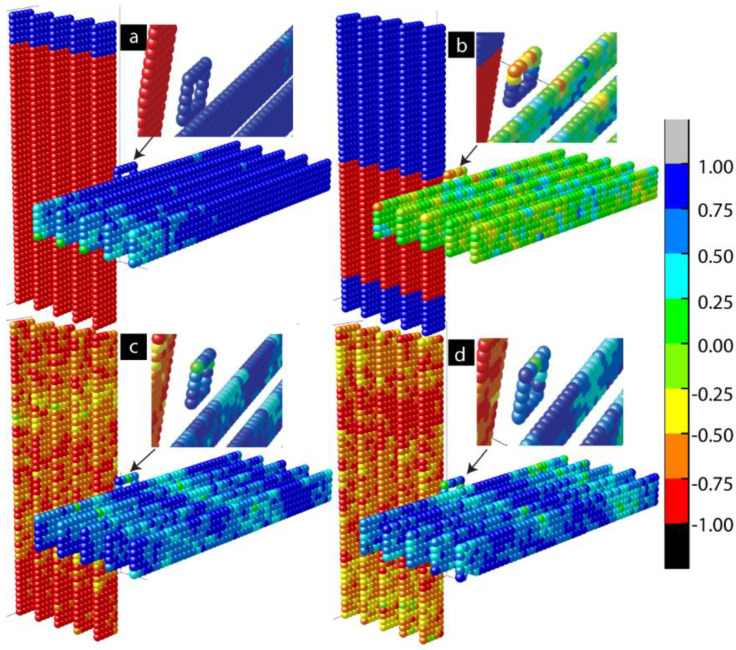
Simulated spatial 3D lattice model of the MTJMSD measured at *kT* = 0.1 (**a**) *A_Lx_* = 1 and *A_Ly_* = 0, (**b**) *A_Lx_* = 0 and *A_Ly_*= 1, and (**c**) *A_Lx_* = 1 and *A_Ly_* = 1 along spin direction on the *x*-axis, and (**d**) *A_Lx_* = 0 and *A_Ly_* = 0.

To understand the actual spin configurations of the left and right FM electrodes, we analyzed the atomic-scale equilibrium moment of the MTJMSD’s Heisenberg model (Figure 2 and Figure 3). Figure 2 shows three 3D spins’ vector intensities along the x, y, and z directions. In 3D atomic schematic representation, the left FM electrode is represented by vertical lattices, while horizontal lattices represent the right FM electrode, and molecules are represented by small squares between the left and right FM electrodes. The color scale bar presented in Figure 2 and Figure 3 represents the normalized magnetic moment. A Monte Carlo simulation (MCS) takes the variable that has uncertainty and assigns it a random seed. The model is then run, and a result is provided. This process is repeated while assigning many different values to the variable in question. Once the simulation is complete by energy minimization, the equilibrium state magnetic moments are averaged together to provide an estimate. As a result, the settlement of the magnetic spins is always arbitrary along the x, y or z spin direction in the absence of anisotropies, as illustrated in Figure 2. In this particular situation, the spins of the magnetic atoms settled in the z direction. The settlement of the magnetic spin direction is completely random unless we provide the same seed or apply the anisotropies during the simulations. The closeness of the color corresponding to the magnetic moment of the molecules and the first right ferromagnetic electrode occurred because the molecules created strong ferromagnetic coupling with the right electrode (*J_mR_*  =  1). On the other hand, the complete color contrast of the molecules with the left ferromagnetic electrode occurred because the molecule created antiferromagnetic coupling with the left electrode (*J_mL_*  =  −1).

Figure 3a represents the 3D lattice model along the x spin direction when out-of-plane magnetic anisotropy *A_Lx_* = 1. Anisotropy caused the multiple magnetic domains of opposite spins that appeared on the left ferromagnetic electrode (Figure 3a). These domains represent the different magnetic phases. With the application of strong out-of-plane anisotropy, a dominant magnetic phase appeared on the left electrode. A residual secondary magnetic phase of opposite spins also appeared, as shown in Figure 3a. The effect of anisotropy appeared on the left ferromagnetic electrode and transferred to the right ferromagnetic electrode via molecular channels. As a result, ordered magnetic spins states appeared on the right electrode despite not having any anisotropies on the right FM electrode (*A_Rx_ = A_Ry_* = 0). The spin orientation of the right FM electrode was opposite to that of the left FM electrode, despite the spin stabilization that happened on the right FM electrode due to the left FM electrode. The molecules maintained the antiferromagnetic couplings with the left FM electrode, represented by opposite spin colors (red and blue for the left FM electrode and molecules, respectively). Since the molecular exchange coupling was transferring the impact to the right FM electrode via molecular channels, the spin orientation of the right FM electrode aligned itself to the molecules’ spin directions. The application of in-plane anisotropy (*A_Ly_* = 1) caused multiple magnetic phases of opposite magnetic spins, as shown in Figure 3b. Unlike out-of-plane anisotropy, there was no dominant magnetic phase. Molecular channels were connected on the left electrode in the boundary region of two phases of opposite spins, as shown in Figure 3b. As a result, the effect of anisotropy appearing on the left electrode could not be transferred to the right electrode. When *A_Lx_ = A_Ly_* = 1, the competing impact of in-plane and out-of-plane anisotropies were observed on the electrode, as shown in Figure 3c,d. When we carefully observed the 3D lattice, Figure 3c, slightly more red spots appeared on the left electrode. This is because the net magnetic moment caused by the dominant magnetic phase on the left electrode was not destroyed due to the competing impact.

**Figure 4:**
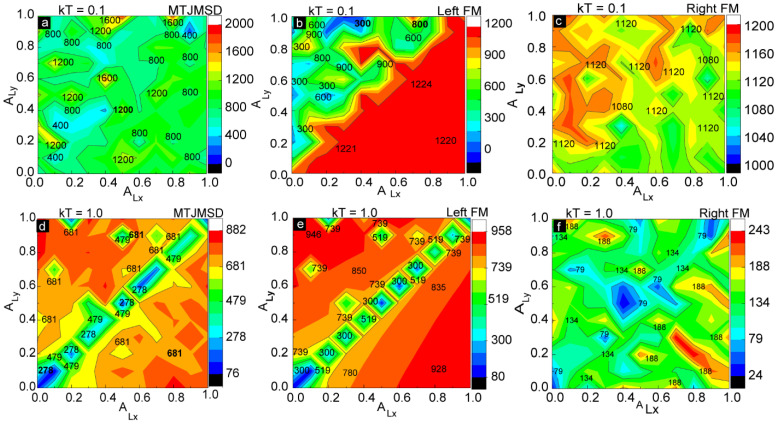
Magnetic moment as a function of in-plane (*A_Ly_*) and out-of-plane (*A_Lx_*) anisotropies at *kT* = 0.1 for (**a**) MTJMSD, (**b**) left ferromagnetic electrode, and (**c**) right ferromagnetic electrode. Magnetic moment as a function of in-plane (*A_Ly_*) and out-of-plane (*A_Lx_*) anisotropies at *kT* = 1 for (**d**) MTJMSD, (**e**) left ferromagnetic electrode, and (**f**) right ferromagnetic electrode.

We also investigated the effect of thermal energy (*kT*) on the MTJMSD for various combinations of anisotropies. Figure 4 represents the magnitude of the magnetic moment measured continuously as a function of anisotropy at constant thermal energies. We varied the thermal energy *kT* from 0.1 to 1. Thermal energy *kT* = 0.1 was near room temperature with the assumption that the Curie temperature of the MTJMSD varied with the FM electrode from 300 °C to 800 °C. Figure 4a represents the contour plot for the magnetic moment of the MTJMSD as a function of *A_Lx_* and *A_L_*_y_ measured at *kT* = 0.1. It is difficult to analyze the magnetic moment of the overall device without understanding the behavior of individual ferromagnetic electrodes. It is also very challenging to identify the regions with in-plane and out-of-plane magnetic phases that are natural outcomes of in-plane and out-of-plane easy-axis anisotropies, respectively [29]. Therefore, we first focused on analyzing the effects on an individual ferromagnetic electrode. Figure 4b represents a plot for the magnetic moment of the left ferromagnetic electrode as a function of *A_Lx_* and *A_Ly_* measured at *kT* = 0.1. It is interesting to note that the magnetic moment of the left FM electrode remains high, varying from 1200 to 1250, for the situation whereby *A_Lx_* ≥ *A_Ly_*. *A_Lx_* stabilized the out-of-plane magnetic direction, represented by the red region on the lower right of the contour diagram (Figure 4b). Out-of-plane anisotropy (*A_Lx_*) caused the formation of a big, single magnetic domain of the same magnetic spin orientation. The big magnetic domain represented a single magnetic phase and was responsible for maintaining a higher magnetic moment along the out-of-plane x direction. In this case, where *A_Lx_* is dominant, the magnetic domain’s direction will be parallel or antiparallel to the out-of-plane x direction. Higher magnetic moments due to the single magnetic domain were consistently observed in the 3D lattice model, as shown in Figure 3a. In the diagonal region, when *A_Lx_* = *A_Ly_*, the magnetic moment of the left ferromagnetic electrode is slightly lower than when *A_Lx_* ≥ *A_Ly_* and remains nearly constant, as illustrated by the orange stripe in Figure 4b. The smaller values of magnetic moments are due to the multiple magnetic phases of opposite spins that appeared in the left ferromagnetic electrodes due to the application of *A_Ly_*. It was also anticipated that the left FM electrode would switch from the out-of-plane to in-plane magnetic direction for *A_Lx_* ≤ *A_Ly_*. The formation of multiple magnetic phases of opposite spins on the same left ferromagnetic electrode due to the application of in-plane anisotropy (*A_Ly_*) is also illustrated in Figure 3b. However, in this case, whereby *A_Ly_* is dominant, the magnetic domain’s direction will be parallel or antiparallel to the in-plane *y* direction.

The magnetic moment of the right FM electrode was relatively high compared to that of the left ferromagnetic electrode since we had not applied any anisotropies to the right ferromagnetic electrode (Figure 4c). However, when we carefully observed the contour plot, there was a general trend in the values of the magnetic moment. For the right ferromagnetic electrode, the magnetic moment was lower in the region *A_Lx_* ≥ *A_Ly_* compared to that of the region *A_Lx_* ≤ *A_Ly_*. As we discussed in Figure 4b, the effect of molecular exchange coupling from the left ferromagnetic electrode can transfer to right electrode via molecular channels. This molecular exchange coupling was responsible for creating moderately aligned magnetic spins in the right electrode even if we did not apply magnetic anisotropies to the right electrode (Figure 4c). Most importantly, molecular coupling played major role in setting the magnetic spin direction on the right FM electrode in accordance with the left FM electrode. The right FM electrode magnetization will be in-plane or out-of-plane based on what easy-axis anisotropy is dominating the left electrode. In essence, the role of strong antiferromagnetic molecular coupling is to set the FM electrode spin orientation opposite to the spin orientation on the left FM electrode. The diagonal region had small variations in its magnetic moment, varying from 1080 to 1120. The magnetic moment of the MTJMSD was the overall sum of the magnetic moments of the left ferromagnetic electrode, the right ferromagnetic electrode, and the molecules.

As the temperature increased, thermal energy started to annihilate the magnetic domains. Here, we discuss the magnetic moments of the MTJMSD (Figure 4d), the left electrode (Figure 4e), and the right electrode (Figure 4f), respectively, measured at kT = 1. In the diagonal region (the region with *A_Lx_* ≈ *A_Ly_*), the in-plane and out-of-plane anisotropy had a competing effect. As a result, the region had a small net magnetic moment value compared to both the *A_Lx_ ≤ A_Ly_* and *A_Lx_* ≥ *A_Ly_* regions. It is noteworthy that *A_Lx_* ≈ *A_Ly_* represents the case whereby four directions are possible, and this scenario, is similar to when no anisotropy is active. As an analogy, zero force on a point is equivalent to equal and opposite forces on the same point. High temperature annihilated the magnetic phases of opposite magnetic spins along in-plane y and out-of-plane x easy axes. Therefore, unlike for *kT* = 0.1, the magnetic moments were nearly symmetric in both the *A_Lx_ ≤ A_Ly_* and *A_Lx_* ≥ *A_Ly_* regions, as shown in Figure 4d. Because of high thermal agitation, molecular exchange coupling could not transfer from the effect of anisotropy on the left electrode to the right electrode via molecular conducting channels (Figure 4f). The right electrode without anisotropy changed from a ferromagnetic to a paramagnetic state after increasing the thermal energy close to curie temperature (Figure 4f). Therefore, magnetic spins on the right electrode were randomly oriented. The magnetic moment of the left FM electrode (Figure 4e) was significantly more than that of right FM electrode (Figure 4f) for *kT* = 1. It is interesting to note that magnetic spins were still in the moderately ordered state even at Curie temperature (*kT* = 1.0) because of in-plane and out-of-plane anisotropies. However, the overall magnetic moment of the MTJMSD (Figure 4d) was less than that of the left electrode (Figure 4e) due to the irregular orientations of the magnetic spins at high thermal energy.

**Figure 5:**
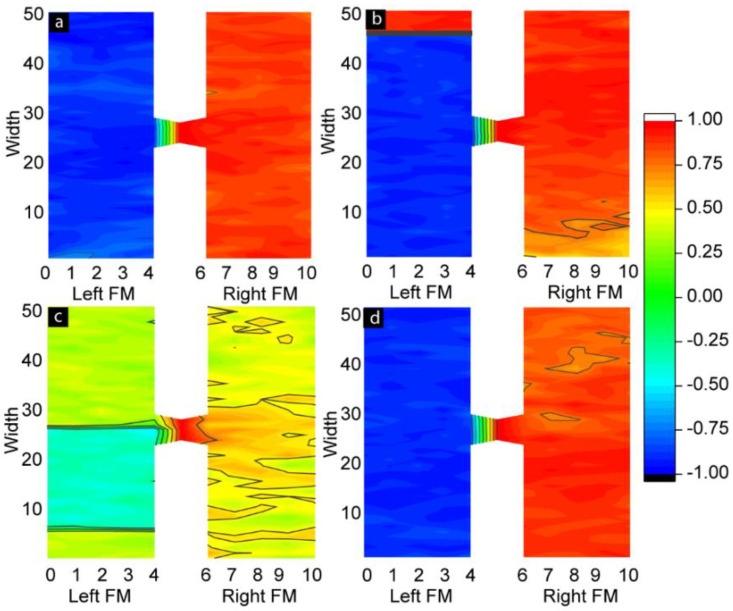
Spatial correlation *(SC*) factor contour plots of MTJMSD at *kT* = 0.1 with (**a**) *A_Lx_* = *A_Ly_* = 0, (**b**) *A_Lx_* = 1 and *A_Ly_* = 0, (**c**) *A_Lx_* = 0 and *A_Ly_* = 1, and (**d**) *A_Lx_* = *A_Ly_* = 1.

We further investigated the length scale of different phases in ferromagnetic electrodes and the spatial correlation between molecular spins and FM electrodes (Figure 5). To quantify the correlation of spins between molecules and atoms in different layers of the ferromagnetic electrodes in the presence of in-plane and out-of-plane anisotropies, we studied the customized spatial correlation (*SC*) factor. The *SC* is the dot product between the average molecular spin vector and the spin vectors in each atomic row of two ferromagnetic electrodes. The equation used to calculate the *SC* is as follows (Equation (1)):

(1) $$SC=(S_{m}\vec{x}+S_{m}\vec{y}+S_{m}\vec{z}).\;(S_{FM}\vec{x}+S_{FM}\vec{y}+S_{FM}\vec{z})$$

A positive *SC* represents parallel alignment of the spins of ferromagnetic atoms with the spins of molecules. A negative *SC* represents antiparallel alignment of the magnetic moment of atoms of the left and right ferromagnetic electrodes with molecular spins. The magnitude of the *SC* suggests the strength of the correlation between the molecule and FM electrode layers. The *SC* contours shown in Figure 5 correspond to the cases of anisotropy shown in the 3D lattice plots in Figure 3. Here, Figure 5a is for *A_Lx_ = A_Ly_ =* 0, Figure 5b for *A_Lx_* = 1 and *A_Ly_* = 0, Figure 5c for *A_Lx_* = 0 and *A_Ly_* = 1, and Figure 5d for *A_Lx_* and *A_Ly_* = 1. When *A_Lx_* = 0 and *A_Ly_* = 0, the spin states of two ferromagnetic electrodes are highly correlated with the spin states of the molecules. Molecule-induced strong antiferromagnetic coupling forced the left and right FM electrodes to assume antiparallel states (Figure 5a and Figure 2c). The atomic spins of the left ferromagnetic atoms were negatively correlated with the molecular spins, while the atomic spins of right ferromagnetic electrodes were positively correlated with the molecular spins. These correlations were expected in the MTJMSD Heisenberg model, since molecules were antiferromagnetically and ferromagnetically coupled with the left and right FM electrodes, respectively. In the absence of anisotropies, the antiferromagnetic coupling of molecules with electrodes was dictated by the *J_mL_* = −1 and *J_mR_* = 1 values. It is noteworthy that the segments of the molecules adjacent to the ferromagnetic electrodes tended to align their spins in strong correlation with the spins of the ferromagnetic electrodes, as shown in Figure 5a,b,d. When the molecular conducting channels were directly on the magnetic phase transition region, multiple magnetic spins were also appeared on the molecular spin states, as shown in Figure 3b and Figure 5c. The domain wall width of multiple magnetic phases also depends upon the anisotropy, which is reported elsewhere. In-plane anisotropy causes the formation of multiple magnetic phases of opposite magnetic spins. However, the high value of *A_Ly_* caused the formation of a dominant magnetic domain on the left ferromagnetic electrode, represented by the red domain color in Figure 5b. The magnetic spins of this dominant region are negatively correlated with the molecular spins, and the region stood up to the 47th atomic layer of the left ferromagnetic electrode. The spatial correlation factor is ~−0.8, as shown in Figure 5b.

A second magnetic domain stands on the 48th, 49th, and 50th atomic layers on the same left ferromagnetic electrode. This domain not only has opposite magnetic spins compared to the dominant magnetic region but is also positively correlated with the molecular magnetic spins, with an equal magnitude of the autocorrelation factor but with the opposite sign, i.e., ~0.8. As discussed previously, out-of-plane anisotropy caused the multiple magnetic phases on the left ferromagnetic electrode (Figure 5c). From atomic layers 0 to 8, the magnetic spins are positively correlated with the molecular spin, with a correlation factor of ~0.3. From layers 8 to 26, the magnetic spins are negatively correlated with the spin of the molecules, with a correlation factor around −0.35. From layers 27 to 50, the magnetic spins are again positively correlated with the molecular spins, with a correlation factor of ~0.3. It is worth mentioning not only that anisotropy creates different magnetic phases of opposite spins, but also that these phases have spins correlated with molecular spins of equal magnitude but with opposite spin orientations. The right ferromagnetic electrode for this case stabilized in a completely random direction (Figure 5c). This is due to the anisotropy effect being unable transfer from the left to the right ferromagnetic electrode via the molecular conducting channel. This is because the molecular conducting channels fall in the region of phase transition. As a result, molecular spins are positively correlated with the spins of an electrode and negatively correlated with another electrode. In the present case, when both anisotropies existed, molecular spins were positively correlated with the magnetic spins of the right ferromagnetic electrode, while they were negatively correlated with the magnetic spins of the left electrode (Figure 5d). The left electrode exhibited a single phase, unlike the appearance of multiple phases observed for unequal in-plane and out-of-plane anisotropies.

## 3. Materials and Methods

### 3.1. Experimental Observations

We experimentally produced pillar-shaped MTJs to investigate the effect of differences in multilayer electrodes on the equilibrium properties of MTJMSDs. In our prior work, we described the process of MTJ fabrication and the method of transforming it into an MTJMSD by bridging molecules along the exposed side between two ferromagnetic electrodes [21]. A pillar-shaped MTJMSD brings distinctive advantages in that the ferromagnetic electrode is confined within the perimeter of the tunnel junction area and the molecular junctions. Hence, no interference or impact of ferromagnetic electrodes beyond the junction area will occur. The MTJs were patterned and deposited on silicon substrate with a ~300 nm silicon dioxide layer. Each of the ~7000 cavities for producing MTJ pillars were photolithographically defined to have a ~25 µm^2^ area. All the MTJ layers were sequentially deposited in the cavities. The bottom electrode was deposited as a bilayer of ~5 nm cobalt (Co) and 5 nm NiFe. A ~2 nm tantalum seed layer was used for promoting adhesion between the Co and silicon dioxide insulating layers. In the photoresist cavity, sequentially, a 2 nm thick alumina (AlOx) and a ~10 nm thick NiFe top electrode were deposited. Utilization of the same photoresist cavity for all depositions ensured that the bottom FM electrode, ~2 nm AlOx, and the top FM electrode had the exact same lateral dimensions; this provision ensured that the minimum physical separation between the top and bottom electrode would be equal to the insulating thickness along the exposed side edges. The photoresist could be easily removed during the liftoff process to provide clean edges for bringing the molecules of interest in the contact of two metal electrodes. Liftoff was performed to remove excess materials and produce a Ta/Co/NiFe/AlOx/NiFe MTJ with an exposed side. Along the exposed side edges, organometallic molecular clusters (OMCs) or Single Magnetic Molecules (SMMs) [30] were bridged across the AlOx to complete the MTJMSD fabrication. In-depth details about OMC properties in an as-produced state are published elsewhere [30,31]. We utilized an electrochemical process for molecular self-assembly that is known to produce good, quick metal–thiol bonding. The OMCs possessed a cyanide-bridged octametallic molecular cluster, [(pzTp)Fe^III^(CN)_3_]_4_[Ni^II^(L)]_4_[O_3_SCF_3_]_4_ [(pzTp) = tetra(pyrazol-1-yl)borate; L = 1-S(acetyl)tris(pyrazolyl)decane] chemical structure. The internal exchange coupling between metallic ions in the OMCs exhibited an S = 6 spin state in the bulk powder form at <10 K. It is extremely challenging to determine the actual OMC spin state when covalently bonded between two ferromagnetic electrodes in an MTJMSD. However, room temperature observations of the spin-photovoltaic effect [32], current suppression [33], and other phenomena [23] assert that OMCs could maintain a net magnetic spin state at room temperature.

**Figure 6:**
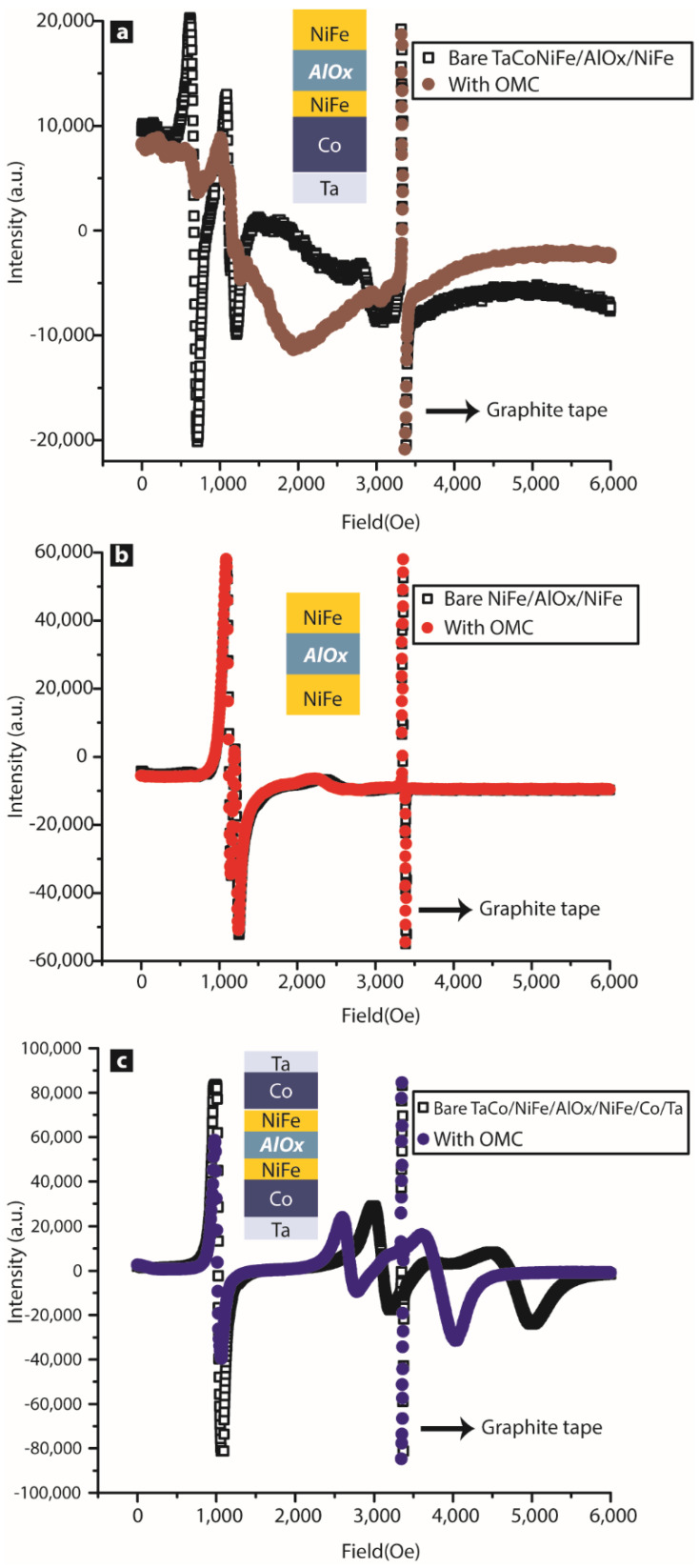
FMR study before and after OMC treatment of ~7000 MTJs/sample with (**a**) Ta/Co/NiFe/AlOx/NiFe, (**b**) NiFe/AlOx/NiFe, and (**c**) Ta/Co/NiFe/AlOx/NiFe/Co/Ta thin-film configuration.

As shown in Figure 6a, OMCs significantly impacted the FMR modes of Ta/Co/NiFe/AlOx/NiFe MTJs. Acoustic mode (bigger peak) and optical mode (smaller resonance peak) on bare MTJ pillars were absent after bridging the OMC channels. OMCs produced strong exchange coupling between two FM electrodes [21]. The bottom electrode containing cobalt was magnetically harder than the NiFe electrode in the present case. Our prior work demonstrated a difference in NiFe and Ta/Co/NiFe electrodes and multiple pieces of experimental evidence showing that OMC produced unprecedented strong inter-electrode antiferromagnetic coupling [21]; we are unsure if spin fluctuations contributed to enhancing the impact of molecular channels like in previous studies [14]. Interestingly, the same OMC molecule did not produce a noticeable impact on MTJ pillars with NiFe/AlOx/NiFe (Figure 6b). In this case, NiFe possessed an in-plane easy axis. This MTJ sample with identical NiFe electrodes also showed acoustic- and optical-mode positions very close to each other compared to the case shown in Figure 6a. In the third case, we treated Ta/Co/NiFe/AlOx/NiFe/Co/Ta CoNiFe pillars with OMCs. The role of Co in this case was to produce out-of-plane easy-axis anisotropy and increase the overall magnetic hardness (coercivity) of the FM electrodes. Interestingly, this sample with both harder FM electrodes was impacted by OMCs (Figure 6c). The acoustic mode amplitude decreased due to the establishment of OMC channels. However, the optical mode (low-amplitude mode) shifted towards the acoustic mode (Figure 6c). We do not have a clear understanding of the mechanism behind this observation and further work is needed to obtain a better understanding. However, the main conclusions of these experimental studies are as follows: (a) OMC impact dramatically differs in MTJs with ferromagnetic electrodes of different magnetic hardness. (b) Ta/Co/NiFe also exhibited an FMR response in the out-of-plane magnetic field due to the presence of Co, whereas NiFe only responded to the in-plane field during the resonance study. This means that OMCs’ strong response in MTJs occurs when at least one FM electrode possesses an out-of-plane anisotropy. (c) OMCs severely impacted the original FM electrodes and transformed them into different materials. Hence, the resultant MTJMSD is expected to have different top and bottom electrode magnetic anisotropy in this multilayer state.

**Figure 7:**
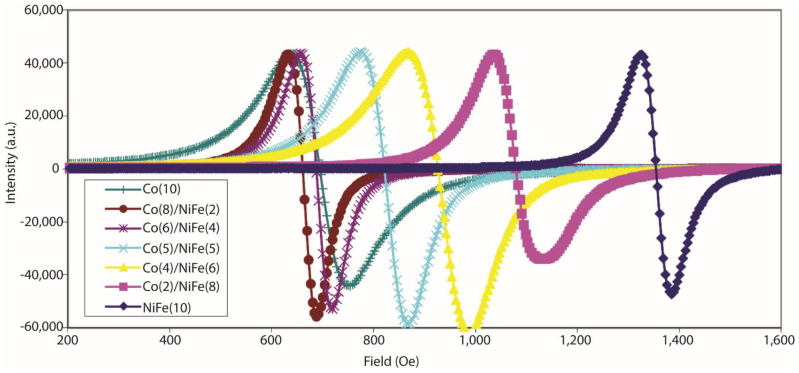
FMR study of Co/NiFe 10 nm thick bilayer with thickness of Co varying from 0 to 10 nm.

We also conducted an FMR study on bilayer ferromagnetic thin films with variable Co and NiFe composition (Figure 7). It is noteworthy that for 10 nm Co and the bilayer containing a Co (8–6 nm)/NiFe (2–4 nm) configuration, the responses were quite similar (Figure 7). This means that the Co/NiFe bilayer with ≥6 nm Co will be dominated by the out-of-plane easy axis for magnetization. The bilayer with ≤5 nm Co thickness starts drifting towards a NiFe-dominated response that is governed by the in-plane easy axis. This FMR study does not intend to provide a quantitative analysis of anisotropy in bilayers. This study mainly suggests the wide range of possibilities when two easy axes are present in the same magnetic electrode.

**Figure 8:**
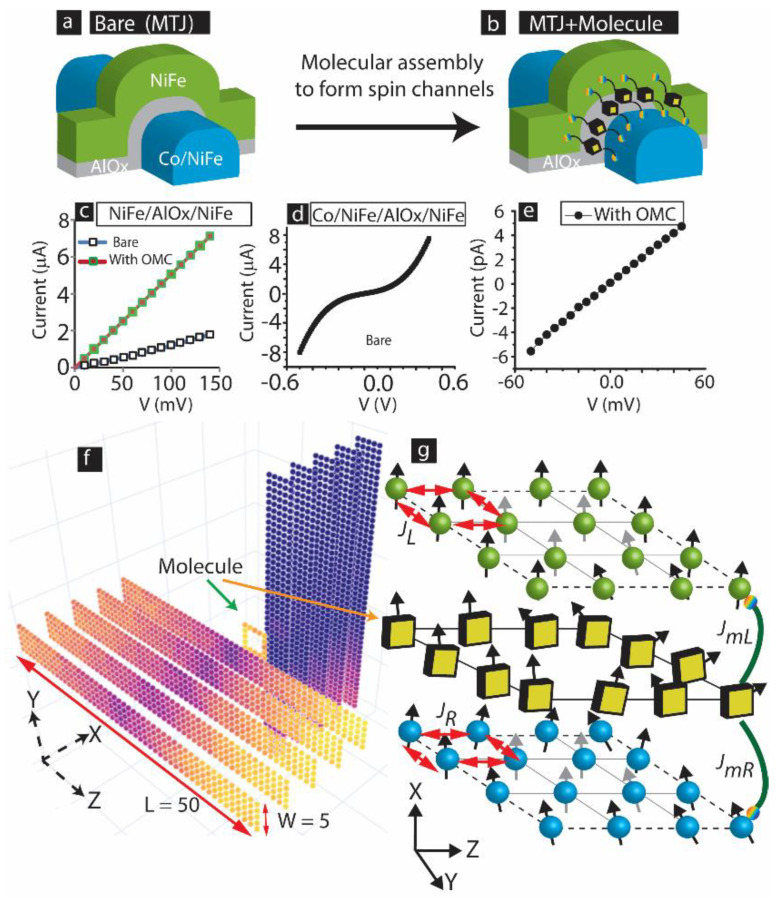
3D sketch of cross-junction-shaped Magnetic Tunnel Junction (**a**) before and (**b**) after connecting molecular channels between two ferromagnetic electrodes. (**c**) NiFe/AlOx/NiFe I-V before and after interacting with molecules. I-V of Co/NiFe/AlOx/NiFe (**d**) in bare state and (**e**) after interacting with OMC molecules. (**f**) A 3D atomic model of molecular device analogous to MTJMSD shown in panel (**b**). (**g**) Description of coupling energy around molecular junction of 3D model shown in panel (**e**).

Magnetic electrodes with multiple anisotropies are a strong contender for developing novel devices and systems patterned in different forms. Cross-junction device architecture has been envisioned for MRAM application [34]. In cross-junction geometry, magnetic electrodes are expected to extend beyond the junction. We extensively studied a cross-junction-shaped MTJMSD with a 10 nm thick Co/NiFe bilayer electrode as the bottom electrode and a ~10 nm thick NiFe top electrode. Our prior research produced multiple pieces of evidence showing OMCs’ dramatic impact on the transport and optical properties of FM electrodes [23,33,35]. MTJMSD cross-junction-shaped device geometry will need extended electrodes (Figure 8a) around the junction to establish the connection between the molecular junction and the outer world (Figure 8b). We observed that OMC molecules responded very differently for NiFe/AlOx/NiFe vs. Co/NiFe/AlOx/NiFe. In the case of NiFe/AlOx/NiFe, charge transport simply increased after connecting OMC channels along the edges (Figure 8c). On the other hand, cross-junction-shaped MTJs with Co/NiFe/AlOx/NiFe resulted in non-linear tunneling in the bare state (Figure 8d) and stabilized to a suppressed current state ~6 orders of magnitude lower at room temperature (Figure 8e). The difference in the transport properties of NiFe/AlOx/NiFe and Co/NiFe/AlOx/NiFe after OMC treatment (Figure 8c–e) resembles the FMR response in Figure 6a,b. It is apparent that a slight difference in electrode composition yielded a dramatic difference in OMC response. In our prior work, OMCs generally created strong antiferromagnetic coupling between the Co/NiFe and NiFe electrodes. Device fabrication details and other experimental information about cross-junction-shaped MTJMSDs are published elsewhere [32].

Conducting a comprehensive study of the wide range of anisotropy magnitudes in one electrode in the cross-junction form of an MTJMSD is a daunting task via conventional DFT or micromagnetic methods. Hence, we focused on an MCS study that enables variation in cross-junction-shaped device geometry that involves thousands of atoms for computation using desktop lab computers. To encompass a wide range of possible magnitudes of the magnetic anisotropies of the two electrodes in the MTJMSD, we varied the in-plane easy-axis anisotropy and the out-of-plane easy-axis anisotropy parametrically. We envisioned that each combination may represent a new case in a futuristic experimental study and may be understood with the help of the MCS study discussed here.

### 3.2. Computational Methodology

This manuscript mainly focuses on a Monte Carlo Simulation (MCS) study of an MTJMSD. We varied two easy-axis anisotropies in only one FM electrode to make the MCS study relevant to the experimentally studied cross-junction-shaped MTJMSDs with one bilayer FM electrode. We also focused on only one FM electrode to extend the insights shared in our recent publications about the impact of a single easy-axis direction in one FM electrode [36]. In our study, only the right FM electrode was isotropic, but in-plane and out-of-plane easy-axis anisotropies were applied to the left FM electrode. All other parameters, except thermal energy (*kT*), which could impact the overall magnetic properties of the MTJMSD, were kept constant during the MCS. To be consistent with the experimental scenarios, we adopted a case whereby molecules produced strong antiferromagnetic coupling with one FM electrode and ferromagnetic coupling with another FM electrode [21]. Magnetic tunnel junction (MTJ) with cross-junction geometry is shown in the bare state in Figure 8a, and with molecules on the edges in Figure 8b. The schematic description of the dimension of the MTJMSD, including the spin orientation of molecules and FM atoms, are described elsewhere [21]. We designed a 3D Heisenberg model to represent cross-junction-shaped devices (Figure 8f). The Hamiltonian for computing the MTJMSD’s energy during the MCS is shown in Equation (2).

(2) $$\begin{array}{c} E=-J_{L}\left(\sum_{i\in L}{\vec{S}}_{i}{\vec{S}}_{i+1}\right)-J_{R}\left(\sum_{i\in R}{\vec{S}}_{i}{\vec{S}}_{i+1}\right)-J_{mL}\left(\sum_{i\in L,i+1\in mol}{\vec{S}}_{i}{\vec{S}}_{i+1}\right)-J_{mR}\left(\sum_{i-1\in mol,i\in R}{\vec{S}}_{i-1}{\vec{S}}_{i}\right)-A_{Lx}\left(\sum_{i\in L}{\vec{S}}_{i}^{2}\right)-\;\\ A_{Ly}\left(\sum_{i\in L}{\vec{S}}_{i}^{2}\right) \end{array}$$

The size of the MTJMSD Heisenberg model in this MCS study was confined in a box of *H × W × L* = 11 × 50 × 50 (volume in atomic units). Here, *H*, *W*, and *L* are atomic height, width, and length, respectively. In this model, FM electrodes measure 5 × 5 × 50, and molecules are represented by a 5 × 5 square with an empty interior, as shown in Figure 8f. The empty interior represents the AlOx-like insulator needed in MTJMSDs to ensure molecule-scale spacing between the two FM electrodes. In Equation (1), *S_i_* represents the spin of FM atoms and molecules. The *S_i+_*_1_ and *S_i_*_−1_ symbols represent the nearest neighbors with respect to spin at the *i*th site. The Heisenberg coupling across the ferromagnetic atoms of left and right electrodes, represented by *J_L_* and *J_R_*, always kept to their maximum values, i.e., *J_L_* = *J_R_* = 1 during the MCS (Figure 8g). Similarly, *J_mL_* represents the Heisenberg coupling of molecules with the atoms of left FM electrode, while *J_mR_* represents the Heisenberg coupling of molecules with the atoms of right FM electrode. To maintain the antiferromagnetic coupling of molecules with left and right FM electrodes, we fixed the values of *J_mL_* = −1 and *J_mR_* = 1, as illustrated in Figure 8g.

In our previous research, we experimentally estimated the strength of molecule-induced exchange coupling [21]. We conducted a temperature vs. MTJMSD magnetic moment study and observed a strong molecule-induced exchange coupling for that breakdown temperature was ~400 K. This temperature is popularly known as the Neel temperature (*T_N_*) in the case of antiferromagnets [21]. The presence of *T_N_* affirmed that OMCs induced net antiferromagnetic coupling between the two FM electrodes. To compute the relative energy needed to break OMC-induced bonding, we compared the OMC-induced *T_N_* with the Curie temperature (*T_c_*) of the NiFe FM electrode. We focused on the NiFe ferromagnet because of the fact that only NiFe FM directly bonded with OMCs. As per the prior literature, NiFe exhibited a *T_c_* of around 800 K [37]. We found that the *T_N_/T_C_* ratio was ~0.5 [21]. Based on the experimental studies, we concluded that OMC-induced antiferromagnetic coupling was in the order of 0.5 times that of the interatomic ferromagnetic exchange coupling strengths. This indirect estimation of the nature and strength of *J_mL_* and *J_mR_* is in accordance with the assumption that the *kT_c_* for an FM corresponds to interatomic exchange coupling [38]. Since this MCS study covers a wide range of molecules and FM electrodes, we have surmised that *J_mL_* and *J_mR_* could be higher than what we computed and selected a magnitude of 1. We also represented molecules as an atomic analog based on our recent research [39]. This simplified representation of molecules makes such MCSs possible. We have shown that beyond a critical value of molecular spin (~0.2), variation in molecule spin state does not dramatically impact long-range ordering on the FM electrodes of an MTJMSD [39].

Under the MCS approach, the energy of the MTJMSD was minimized to reach the equilibrium state. During the simulation, the ambient thermal energy in which the MTJMSD operated was represented by *kT*. The *kT* factor plays critical role in the Metropolis algorithm in producing stable MTJMSD configuration for each combination of simulation parameters. The MCS method has been discussed elsewhere [21]. In the present MCS study, we fixed *kT* at 0.1. In practical terms, *kT* = 0.1 corresponds to the operational temperature, which ranged from 50 °C to 130 °C after accounting for internal computer heating, was close to room temperature. Our analogy is based on the assumption that the Curie temperature of various candidate FM electrodes can vary from 500 °C to 1300 °C [29].

We studied the impact of unidirectional out-of-plane anisotropy along the x direction easy-axis (*A_Lx_*) and in-plane anisotropy along the y direction easy axis (*A_Ly_*) on the left FM electrode. We varied all the possible combinations for *A_Lx_* and *A_Ly_*. We varied the values of *A_Ly_* from no anisotropy (*A_Ly_* = 0) to its maximum value, i.e., *A_Ly_* = 1 for all possible values of *A_Lx_* (from *A_Lx_* = 0 to 1 in steps of 0.1). To keep the discussion generic, the exchange coupling parameters, magnetic anisotropy, and thermal energy are referred to as unitless parameters throughout this computational study.

## 4. Conclusions

This paper discussed the impact of various anisotropy natures on an MTJMSD. We gained the following insights.

1. We experimentally showed that variation in the in-plane and out-of-plane easy axis of ferromagnetic electrodes connected to exactly the same paramagnetic molecular channels yielded dramatically different equilibrium properties.
2. MTJMSDs offer unprecedented opportunities to innovate novel spintronics devices through simple variation in the thin-film electrode layers. Our FMR study showed that a 10 nm thick bilayer of Co/NiFe yielded significant differences in magnetic properties for different ratios of Co and NiFe thickness.
3. A cross-junction-shaped MTJMSD is a strong candidate for cross-bar geometry-dependent logic and memory devices, as proposed in the prior literature. Our initial transport studies with cross-junction-shaped MTJMSDs showed that variation in FM electrode anisotropy yielded a dramatically different response. Our experimental study necessitated the investigation of a wide range of magnetic anisotropies on an MTJMSD. We adopted MCS methodologies due to their distinctive advantage in handling complex MTJMSD requirements.
4. We focused on Monte Carlo Simulation (MCS) to analyze the variation in the magnetic moment as a function of iterations, anisotropies, and thermal energy. During the MCS study, we applied in-plane and out-of-plane anisotropies to the left ferromagnetic electrode while keeping the right ferromagnetic electrode isotropic.
5. We observed that the presence of in-plane anisotropy caused multilayer magnetic phases on the same ferromagnetic electrode of the magnetic tunnel junction (MTJ). These multiple magnetic phases of opposite spins behaved as soft and hard magnetic phases. The strong magnitude of out-of-plane anisotropy resulted in a dominant magnetic phase on the ferromagnetic electrode so that the magnetic moment of the overall MTJMSD was higher for the region *A_Lx_* ≥ *A_Ly_*.
6. The simultaneous application of in-plane and out-of-plane anisotropies starts to negate the overall anisotropy properties. The electrode was completely isotropic when *A_Lx_* = *A_Ly_*. The computationally analyzed magnetic properties of the MTJMSD will provide deep insight into the future experimental study of molecular spintronics and molecular-based magnetic tunneling junction devices.
7. Our experimental studies highlight the unique attributes of MTJMSDs for harnessing molecules as device element. Our MCS study provides a representative understanding of the equilibrium properties of MTJMSDs evolving due to the variations in anisotropies at different thermal energies.

## Data Availability

The data supporting this study’s findings are available within the article. The additional data supporting this study’s findings are available from the corresponding author upon reasonable request.
